# Cutaneous leishmaniasis caused by Leishmania infantum arising within a psoriatic plaque: a case report^؆^

**DOI:** 10.1016/j.abd.2026.501339

**Published:** 2026-04-17

**Authors:** Pieter Bourgeois

**Affiliations:** Department of Dermatology, Universitair Ziekenhuis Antwerpen, Edegem, Antwerpen, Belgium

Dear Editor,

Cutaneous leishmaniasis (CL) is a neglected tropical disease with incidence estimates of 700.000 to 1.2 million cases per year; approximately 95% of those cases occur in the Americas, the Mediterranean basin, the Middle East, and Central Asia.^1^ Typical clinical presentation varies from single granulomatous lesions or ulcerations on the site of inoculation, but can also resemble other dermatological diseases, such as pyoderma gangrenosum and psoriasis.^2^, ^3^ However, CL developing in pre-existing skin lesions has rarely been reported. This report describes the first case of an immunocompetent host with the appearance of CL in a longstanding psoriasis plaque.

A 64-year-old Caucasian man presented to the dermatology department with a painless non-healing wound on his right elbow. He had had psoriasis on this site for 7 years, waxing and waning, and he had noticed some oozing six months before our first consultation, while he was staying in his coastal house south of Valencia, Spain, where he spends several months every winter. He didn't recall any trauma. He had travelled to Cambodia and Vietnam five years before, but had no recent visits to tropical regions. He was otherwise in good general health. Prior to referral to our tertiary centre, a dermatologist treated him empirically with three courses of antibiotics ‒ amoxicillin-clavulanic acid (dose unspecified) and two other regimens of unknown composition ‒ without improvement. Cultures from the wound and purulent material obtained before the first antibiotic course yielded only normal skin flora. The lesion fluctuated in size, occasionally almost closing but never fully healing. At presentation, his sole therapy was topical disinfection with povidone-iodine solution. Aside from long-standing psoriasis, his medical history was unremarkable. His chronic medications consisted of rosuvastatin 20 mg once daily and pantoprazole 20 mg once daily.

On dermatological examination, the right elbow displayed an erythematous, infiltrated lesion with scaling, crusting, slight oozing, and an irregular border, centred within an 8 cm psoriatic plaque (Fig. 1). Classic erythrosquamous psoriatic plaques were present on the extensor aspects of both knees and the other elbow, and also the oil-drop pigmentation of the fingernails was consistent with psoriasis. No hepatomegaly, splenomegaly, or palpable lymphadenopathy was detected on physical examination.

**Fig. 1:**
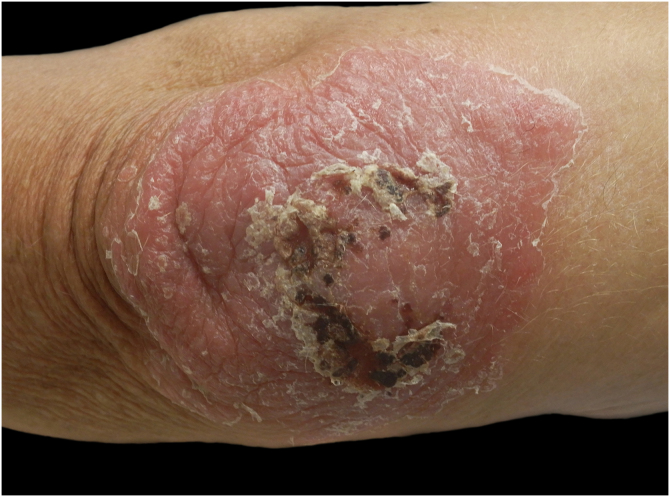
Hematic crusts, ulceration and discrete oozing arising within a psoriatic plaque on the right elbow as the first manifestation of CL caused by L. infantum.

The differential diagnosis included atypical pyoderma gangrenosum, deep cutaneous mycosis, cutaneous leishmaniasis (despite weak epidemiological risk), squamous cell carcinoma, and allergic contact dermatitis.

Ultrasound of the upper arm and right axilla demonstrated reactive lymphadenopathy without hepatosplenomegaly. Routine laboratory tests, including complete blood count and inflammatory markers, were within normal limits.

A punch biopsy from the lesion centre revealed a dense dermal infiltrate of plasma cells and histiocytes forming granulomas; no amastigotes were found. The first PCR determined on a skin sample was positive for Leishmania spp. but lacked species identification. A second biopsy confirmed Leishmania infantum by PCR (Hsp-70 sequencing).^4^

Following confirmation of the diagnosis and identification of the Leishmania infantum subtype, therapeutic options were carefully evaluated. Because the lesion was located within a psoriatic plaque, local therapy ‒ typically considered first-line in Old World CL standard cases ‒ was not deemed appropriate. The patient was therefore admitted for intravenous amphotericin B at a dosage of 5 mg/kg/day for five consecutive days. This regimen has demonstrated efficacy against L. infantum and is more readily accessible in Belgium compared to pentavalent antimonials, which continue to represent the first-line systemic treatment for cutaneous leishmaniasis in many countries.^5^ The crusting and oozing subsided completely within six weeks. However, the original psoriasis lesion persisted; to treat the remaining psoriatic plaques on the right elbow and other body sites, a topical spray of calcipotriol + betamethasone dipropionate was applied once daily for two weeks, then tapered to a maintenance schedule. All psoriasis lesions improved within two months.

CL arising within psoriatic plaques is exceedingly rare. To date, only one comparable case has been reported: a 42-year-old Italian man on long-term methotrexate and adalimumab who developed multiple ulcerations within his psoriatic lesions.^6^ Tumour necrosis factor-α inhibitors are well-recognized risk factors for granulomatous infections, including leishmaniasis.^7^ Our patient was immunocompetent.

CL often mimics a range of dermatoses, but its emergence within pre-existing skin lesions is exceptionally rare, underscoring the uniqueness of this case. Equally noteworthy is the culprit species ‒ Leishmania infantum ‒ traditionally linked to visceral disease yet increasingly implicated in cutaneous infections in Spain, amongst other countries in the Mediterranean region.^8^, ^9^ This trend highlights the importance of including CL in the differential diagnosis of chronic skin lesions in patients with a history of travel to Spain, even in the absence of journeys to classic endemic hotspots.

## ORCID ID

Pieter Bourgeois: 0009-0008-4348-3344

## Financial support

None declared.

## Author' contributions

Pieter Bourgeois: Approval of the final version of the manuscript; design and planning of the study; drafting and editing of the manuscript; critical review of the literature; critical review of the manuscript.

## Research data availability

Does not apply.

## Conflicts of interest

None declared.
